# A model-based circular binary segmentation algorithm for the analysis of array CGH data

**DOI:** 10.1186/1756-0500-4-394

**Published:** 2011-10-10

**Authors:** Fang-Han Hsu, Hung-I H Chen, Mong-Hsun Tsai, Liang-Chuan Lai, Chi-Cheng Huang, Shih-Hsin Tu, Eric Y Chuang, Yidong Chen

**Affiliations:** 1 Graduate Institute of Biomedical Electronics and Bioinformatics, Department of Electrical Engineering, National Taiwan University, Taipei 106, Taiwan; 2 Greehey Children's Cancer Research Institute, The University of Texas Health Science Center at San Antonio, San Antonio, TX 78229, USA; 3 Department of Epidemiology and Biostatistics, The University of Texas Health Science Center at San Antonio, San Antonio, TX 78229, USA; 4 Institute of Biotechnology, Center for Systems Biology and Bioinformatics, National Taiwan University, Taipei 106, Taiwan; 5 Graduate Institute of Physiology, National Taiwan University, Taipei 100, Taiwan; 6 Cathy General Hospital, Taipei 106, Taiwan

## Abstract

**Background:**

Circular Binary Segmentation (CBS) is a permutation-based algorithm for array Comparative Genomic Hybridization (aCGH) data analysis. CBS accurately segments data by detecting change-points using a maximal-*t *test; but extensive computational burden is involved for evaluating the significance of change-points using permutations. A recent implementation utilizing a hybrid method and early stopping rules (hybrid CBS) to improve the performance in speed was subsequently proposed. However, a time analysis revealed that a major portion of computation time of the hybrid CBS was still spent on permutation. In addition, what the hybrid method provides is an approximation of the significance upper bound or lower bound, not an approximation of the significance of change-points itself.

**Results:**

We developed a novel model-based algorithm, extreme-value based CBS (eCBS), which limits permutations and provides robust results without loss of accuracy. Thousands of aCGH data under null hypothesis were simulated in advance based on a variety of non-normal assumptions, and the corresponding maximal-*t *distribution was modeled by the Generalized Extreme Value (GEV) distribution. The modeling results, which associate characteristics of aCGH data to the GEV parameters, constitute lookup tables (eXtreme model). Using the eXtreme model, the significance of change-points could be evaluated in a constant time complexity through a table lookup process.

**Conclusions:**

A novel algorithm, eCBS, was developed in this study. The current implementation of eCBS consistently outperforms the hybrid CBS 4× to 20× in computation time without loss of accuracy. Source codes, supplementary materials, supplementary figures, and supplementary tables can be found at http://ntumaps.cgm.ntu.edu.tw/eCBSsupplementary.

## Background

Copy number alterations (CNAs) are genomic disorders that closely correlate with many human diseases [1]. For instance, 17q23 was found to be a common region of amplification associated with breast cancers with poor prognosis [2], and copy number losses at 13q and gains at 1q and 5p were frequently observed in a prostate cancer study [3]. While some CNAs are well studied, most CNAs and their relation to genetic disorders remain largely unknown. Identifying regions of DNA copy number gains or losses is thus a critical step for studying the pathogenesis of cancer and many other diseases.

Array comparative genomic hybridization (aCGH) is a high throughput and high-resolution technique for measuring CNAs [4,5], and the main purpose of aCGH data segmentation is to detect CNAs precisely and efficiently by utilizing neighboring probes' characteristics. Many algorithms have been proposed for this purpose, such as the Clustering Method Along Chromosomes (CLAC) [6], Hidden Markov Model methods [7,8], and Bayesian segmentation approaches [9,10]. Among these algorithms, Circular Binary Segmentation (CBS) [11] has the best operational characteristics in terms of its sensitivity and false discovery rate (FDR) for change-point detection [12,13].

CBS performs consistently [13] and has been widely used by researchers [14,15], but the major weakness of heavy computational cost prohibits CBS for high-density aCGH microarrays with millions of probes. The original CBS relies fully on a permutation-based maximal-*t *test to detect change-points. Through a recursive cut and test process, reliable results can be derived but require extensive computational burden. Realizing the deficiency, a hybrid version of CBS that mixes the permutations and a mathematical approximation was recently proposed [16]. Along with additional early stopping rules for approximating the significance of change-points, the hybrid CBS can detect change-points in a linear time and provides a substantial gain in speed.

However, further improvements in CBS are still urgently needed. First, the computation time of the hybrid CBS needs to be improved because of the growing density of commercial microarrays. A time consumption investigation of the hybrid CBS revealed that the majority of the computation time, about 94% of total time, was spent on significance evaluation, with the bulk of the time being consumed by permutations (shown in Table 1). This finding indicates that limiting permutations may be necessary. Without the improvement, for example, it might take days to segment 500 samples generated by the most recently completed ovarian cancer study by the Cancer Genome Atlas (TCGA) research network [17]. Second, what the mathematical approximation in the hybrid method provides is an approximation of significance upper bound or lower bound, not an approximation of the significance of change-points itself. As indicated in [16], using an significance upper bound to reject the null hypothesis may result in fewer change-points.

**Table 1:** The percentage of time consumed on each step of segmentation using the hybrid CBS

		Consumed Time	
	
Array	Candidate Location (%)	Significance Evaluation (%)	Edge Effect Correction (%)
Array #10	1.21	88.24	10.56
Array #19	2.32	91.53	6.14
Array #22	1.63	96.21	2.16
Array #28	1.01	95.95	3.05
Array #42	0.87	97.54	1.59
Array #45	0.99	96.43	2.58
Array #48	1.48	98.48	0.04
Array #65	1.11	97.63	1.26
Array #72	1.89	83.92	14.19
Array #78	2.01	91.51	6.48

Ten breast cancer aCGH data were segmented using the hybrid CBS. The time consumed on each of the three steps involved in segmentation, namely, candidate location, significance evaluation, and edge effect correction, was analyzed and listed. Here, significance evaluation was done by a hybrid method and early stopping rules.

In this study, we proposed a model-based version of CBS, termed extreme-value based CBS (eCBS). Instead of evaluating an significance upper bound or a lower bound, eCBS approximates the significance of change-points using a Generalized Extreme Value (GEV) distribution model (eXtreme model). The eXtreme model consists of a set of lookup tables built in advance through simulation. Considering a variety of non-normal aCGH data, we simulated thousands of data without change-points using the Pearson system. The corresponding maximal-*t *distribution was then modeled by the GEV distribution, and the modeling results in the form of the GEV parameters constituted the eXtreme model. Using the eXtreme model, the significance of change-points can be approximated through a table lookup process in a constant time complexity. As a result, permutations are limited and computation time can be significantly reduced. The performance of segmentation in speed and segmentation results using both the hybrid CBS and eCBS were compared via simulation and real data analysis.

## Methods

### Algorithm - Finding Change-Points

Similar to CBS, the newly proposed algorithm, eCBS, detects change-points relying on a sequence of maximal-*t *tests. Before getting into the details of the maximal-*t *test, we first introduce the maximal-*t *statistic as follows. Let *r*_1_,..., *r_N _*be log_2_ ratios of signal intensities indexed by *N *ordered genomic markers. Let *S_i _*= *r*_1 _+ ... + *r_i_*, 1 ≤ *i *≤ *N*, be the partial sums, and let $S_{ij}=S_{j}-S_{i}=\sum_{l=i+1}^{j}r_{l}$. Statistics *T_ij _*are given by [11]

(1) $$T_{ij}=\left(\frac{S_{ij}}{k}-\frac{S_{N}-S_{ij}}{N-k}\right)∕\left(s\sqrt{\frac{1}{k}+\frac{1}{N-k}}\right),$$

where 1 ≤ *i < j *≤ *N*, *s *refers to the standard deviation of *r*_1_,..., *r_N_*, and *k *= *j - i*. Among all values of *T_ij _*derived from all possible permutes of *i *and *j *under consideration, the maximal statistic, *T_max_*, is referred to as the maximal-*t *statistic, and the corresponding locations, *i_c _*and *j_c_*, are termed candidate change-points, which are given by

$$\left(i_{c},j_{c}\right)=\underset{1\leq i<j\leq N}{\text{arg}\;\text{max}}|T_{ij}|,\;1\leq i_{c}<j_{c}\leq N.$$

Based on the maximal-*t *statistic, a maximal-*t *test with two hypotheses (*H*_0_: there is no change-point, *H*_1_: there are change-points locating at *i_c _*and *j_c_*) is formulated. We reject the null hypothesis *H*_0 _and declare locations *i_c _*and *j_c _*as change-points if *T_max _*exceeds a significance threshold.

For a chromosome under consideration, similar to CBS, eCBS detects the regions with equal DNA copy numbers by recursively invoking a change-point function, named "*finding change-points*", in which two ends of a sequential data are first connected and then ternary splits are determined using the maximal-*t *test. The function of finding change-points contains three steps: 1) apply maximal-*t *statistic to locate candidate change-points *i_c _*and *j_c_*; 2) determine whether the candidate change-points are significant or not; and 3) if significant change-points occur, a *t*-test is applied to remove errors near the edges. We refer to these three steps as candidate location, significance evaluation, and edge effect correction.

**Figure 1:**
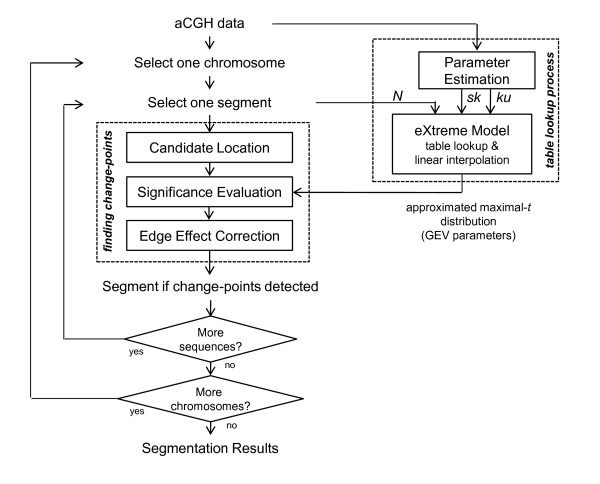
**The block diagram of eCBS**. The block diagram demonstrates how eCBS works. The parameter *N *refers to the number of probes in the sequential data under consideration; the parameters *sk *and *ku *refer to the estimates of skewness and kurtosis, respectively, from the aCGH data.

The implementation of the process for finding change-points in eCBS is basically the same as CBS except the method for significance evaluation. More precisely, in the original CBS, maximal-*t *distribution (distribution of the maximal-*t *statistics under null hypothesis) and the significance of change-points are evaluated using permutations; in the hybrid CBS, the significance of change-points is approximated using a mixed procedure, constituted of permutations, a mathematical approximation, and early stopping rules. In this study, eCBS approximates the significance of change-points using the eXtreme model through a table lookup process. Indexed by the measures of skewness and kurtosis estimated from the array and the number of probes in the sequential data under consideration, the eXtreme model provides an approximation of maximal-*t *distribution in the form of GEV parameters. We will introduce the GEV distribution and the GEV parameters later in the next subsection. We herein demonstrate the implementation of the function - *finding change-points* - in eCBS as Algorithm 1. Please note that the input variables, *gevParams*, represent the approximated maximal-*t *distribution provided by the eXtreme model. Additionally, *gevcdf(T_max_, gevParams)* is a function returning the cumulative distribution function of GEV distribution with parameters *gevParams *at the value *T_max_*. See Figure 1 for the overall concept.

**Algorithm 1 **- finding change-points

change points = *finding change-points (data, gevParams)*

**Input: ***data*: aCGH data to be segmented, 1 × *N *vector

*gevParams*: GEV parameters, *γ*, *σ*, *μ*.

**Output: ***change-points*: a list of change-points

Step:

1. Compute statistics, *T_ij_*, for all possible locations *i *and *j *by Eq.(1);

2. Find candidate change-points *i_c _*and *j_c _*with maximal statistic, *T_max_*;

3. Evaluate the significance of change-points, *p*-value, using

*p-value = 1-gevcdf(T_max_, gevParams)*;

4. Edge effect correction;

5. If change-points are detected, list change-points into *change-points*; also, cut and define new subsegments.

### The Table Lookup Process

Accurate approximations of maximal-*t *distribution using the eXtreme model depend on robust estimators of skewness and kurtosis; incorrect estimates of skewness and kurtosis from aCGH data render the table lookup process incapable of finding correct values. Since the estimation of skewness and kurtosis could be biased due to extremely large values [18], a pre-segmentation process - scanning obvious change-points quickly before segmentation - is selected (see Additional File 1: Supplementary Materials). The pre-segmentation process (see Algorithm 2) is quite similar to formal segmentation, but with lower resolution and no edge effect correction. After subtracting the changes of mean values from copy number amplifications or deletions, skewness and kurtosis can be estimated without bias due to CNAs. Since no permutations are involved, the pre-segmentation process is done in a short time.

**Algorithm 2 **- parameter estimation

[sk, ku] = parameter_estimation(data)

**Input: ***data:* aCGH data to be segmented, 1 × *N *vector

**Output: ***sk*, *ku*: estimates of skewness and kurtosis.

Step:

1. Execute pre-segmentation process without edge effect correction;

2. Discard small segments;

3. Subtract all segments' mean values;

4. Derive measures of skewness and kurtosis after removing segments' mean values.

The table lookup process for deriving approximations of maximal-*t *distribution can be done by sending three indexes, namely, estimates of skewness and kurtosis and the number of probes under consideration, to the eXtreme model. If these indexes do not fall exactly on the table grid, linear interpolation is applied to accomplish the approximation. We apply linear interpolation because the GEV parameters change smoothly with increasing or decreasing values of skewness, kurtosis, and number of probes (shown later). Through the table lookup process, the eXtreme model can provide accurate approximations of maximal-*t *distribution when the number of probes in the sequential data under consideration is not too small (described later). We empirically set the default minimum as 100 probes; when number of probes is less than the minimum, the mixed procedure applied in the hybrid CBS kicks in. Additional improvement in computation time may be achieved using a smaller minimum at the expense of accuracy.

### Simulating aCGH Data Using the Pearson System

**Figure 2:**
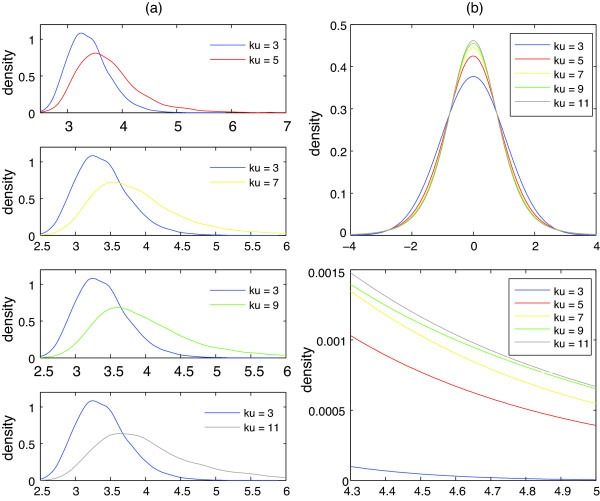
**Tail effect on maximal-***t ***distribution**. (a) The kernel smoothing density of the maximal-*t *statistics derived from 10,000 datasets drawn from the Pearson system (Eq.(2)) with skewness = 0 and various kurtosis. Each dataset contained 250 random numbers. (b) The smoothed probability density functions (PDFs) of the Pearson system with skewness = 0 and various kurtosis. The bottom of Figure 2b illustrates the heaviness of right tails for various kurtosis.

The basic idea to create the eXtreme model, which associates characteristics of aCGH data with maximal-*t *distribution, was to simulate a large number of aCGH data and then model the distribution of maximal-*t *statistics under null hypothesis using the GEV distribution. While a fundamental assumption of aCGH data is Gaussian normality, practical microarray data can be non-normally distributed [19]. Additionally, as we observed in Figure 2, maximal-*t *distribution is quite sensitive to outliers and heavy tails of aCGH data.

Thus, in order to provide accurate modeling results for most aCGH data, we need to consider non-normal properties, namely, the skewness and kurtosis, when generating synthetic datasets.

The Pearson system [20] provides a way to construct probability distributions in which skewness and kurtosis can be adjusted. Without knowing the probability distributions from which aCGH data arose, we hypothesized that, by providing up to 4*^th ^*moments (mean, variance, skewness and kurtosis), the Pearson system is sufficient to simulate a wide range of aCGH data under null condition (no change-points). This hypothesis was clearly hold under our simulated condition by the Kolmogorov-Smirnov Test (KS-test) (see Additional File 1: Supplementary Materials for details of the testing results). Including a set of probability density functions (PDFs), the Pearson system is the solution to the differential equation,

(2) $$\frac{p^{\prime }\left(x\right)}{p\left(x\right)}=\frac{x-a}{b_{2}x^{2}+b_{1}x+b_{0}},$$

where *p *(*x*) is a density function and *a*, *b*_0_, *b*_1_, and *b*_2 _are parameters of the distribution. Correspondences between the distribution parameters and the central moments (*θ*_1_, *θ*_2_, *θ*_3_, *θ*_4_) [21] are denoted as

$$\begin{array}{c} b_{0}=-\left(\theta_{2}\left(4\beta_{2}-3\beta_{1}^{2}\right)\right)∕A,\\ b_{1}=-\left(\sqrt{\theta_{2}}\beta_{1}\left(\beta_{2}+3\right)\right)∕A,\\ b_{2}=-\left(2\beta_{2}-3\beta_{1}^{2}-6\right)∕A, \end{array}$$

where $\beta_{1}=\theta_{3}\text{/}\sqrt{\theta_{2}^{3}}$ denotes the skewness, and $\beta_{2}=\theta_{4}\text{/}\theta_{2}^{2}$ denotes the kurtosis, and $A=10\beta_{2}-12\beta_{1}^{2}-18$.

A MATLAB function,*pearsrnd()*, was applied to generate data with different parameters. Again, since these data were generated under null hypothesis, they contain no change-points. Therefore, the maximal-*t *statistics generated from these data satisfy the null hypothesis. To be specific, we generated tables of data for,

1. The number of probes, *N*, which varies from 10 to 10,000 with intervals of 10, 100, and 1000 for the number of probes within 100, 1000 and 10000, respectively;

2. Skewness, *sk*, selected from -1 to 1 with an interval of 0.1; and

3. Kurtosis, *ku*, selected from 2.6 to 5.6 with an interval of 0.2.

Please note that skewness is 0, and kurtosis is 3 for a normal distribution. The ranges and intervals of the simulation parameters were carefully chosen based on typical estimates of skewness and kurtosis from real aCGH data (see Additional File 1: Supplementary Figure. One can always refine the table resolution when improved computation resources are accessible. Note that the mean and standard deviation of the simulated data were irrelevant to GEV modeling due to the normalization process in Eq.(1), we thus set the mean and standard deviation for simulating aCGH data to be 0 and 1, respectively.

### Modeling Maximal-*t *Distribution Using the GEV Distribution

After simulating aCGH datasets and the subsequent maximal-*t *statistics, we modeled maximal-*t *distribution by the GEV distribution with parameters (described later), *γ*, *σ*, and *μ*, using a maximal likelihood method [22]. Please note that the GEV parameters were derived from the maximal-*t *statistics, not directly from the simulated datasets. The modeling process was done using a MATLAB function (statistical toolbox), *gevfit()*, a maximum likelihood estimator of the GEV parameters. As a result, tables of modeling information (one table for each GEV parameter), indexed by skewness, kurtosis and the number of probes, were generated and saved in the eXtreme model on which eCBS was based.

We briefly go through the GEV distribution and the GEV parameters as follows. For a sequential independent and identically distributed (*i.i.d*.) random variables, *t*_1_,..., *t_n_*, the Fisher-Tippet theorem states that after proper normalization, the maximum of these random variables, *t_max _*= max (*t*_1_,..., *t_n_*), converges in distribution to one of three possible distributions: the Gumbel distribution, the Fréchet distribution, or the Weibull distribution. The three distributions listed above are unified under the Generalized Extreme Value (GEV) distribution [23,24]. The density function of the GEV distribution with parameters (*μ*, *σ*, *γ*) is given by

$$f\left(t_{max}\right)=\left\{\begin{array}{c} \sigma^{-1}w^{-\frac{1}{\gamma }-1}e^{-w^{-\frac{1}{\gamma }}},\;w>0,\text{ for }\gamma \neq 0\\ \sigma^{-1}e^{-z}e^{-e^{-z}},\forall t,\text{ for }\gamma =0 \end{array}\right),$$

where *w *= (1 + *γz*), *z *= (*t_max _- μ*)*/σ*, *γ *is the shape parameter, *σ *is the scale parameter (*σ >*0), and *μ *is the location parameter.

In our setting, the statistics *T_ij _*derived from the quotient in Eq.(1), instead of being a sequential *i.i.d*. random variables, form a random field spatially correlated in the *i *- *j *plane. However, it is not difficult to show that the covariance between any two random variables in the *i *- *j *plane, defined by *T_ij_*, is small when the distance between them or the number of probes, *N*, is large. Furthermore, as shown in [25,26], under certain conditions (independence when sufficient apart and non-clustering), Extreme Value Theory (EVT) can be established for a dependent process by constructing an independent process with the same distribution function. The rigorous mathematical derivation of the distribution of *T_max _*(the maximum among all *T_ij_*) could be considerably difficult due to the complex dependency and beyond the scope of this paper, we thus simply assume that the distribution of *T_max _*(i.e., maximal-*t *distribution) can be modeled by the GEV distribution when *N *is properly large, taking on different sets of parameters than that of GEV distribution for *i.i.d*. random variables.

### Simulation for Performance Validation

We validated the performance of eCBS via simulation. Two simulation models, similar to previous models used by Olshen *et al*. (2004) [11], were applied to eCBS. The first model contains 150 probes (*N *= 150) and four change-points located at 50, 70, 80 and 100. Log_2_ ratios within each segment were randomly generated by normal distribution or *X *~ *N*(*m_i_*, *v*^2^), where *v *is the standard deviation and *m_i _*is the mean value of the *i*-th segment, which was set to be 0, *cv*, *-cv*, *cv*, and 0, respectively. Parameter *c *controlled the alteration amplitudes, and cases with *c *= 2, 3, and 4 were tested. The second model contains 1,500 probes (*N *= 1,500) and one change-point near the edges or two change-points in the center of the chromosomes. Data were generated exactly the same as in the first model, but the change-point locations and amplitudes were controlled by *m_i _*= *cvI*, where *I *is an indicator function, which equals 1 for segments between *l < × <*(*l *+ *k*) and 0 otherwise. Parameter *k *refers to the width of the variation, and *l *refers to the location of the variation.

ROC curves were further used to evaluate the power of detecting change-points in the simulated data drawn from the Pearson System. A segment of copy number variations, 15 probes in width (*k *= 15), was embedded near the edges (*l *= 0) of every chromosome with 1,500 probes (*N *= 1,500). Successful detection of change-points from these data was defined as sensitivity. Cases without copy number variations were also tested for deriving specificity. Different settings of variation amplitudes, skewness, and kurtosis were tested, and the performance of the hybrid CBS and eCBS were compared using ROC curves.

### Real aCGH Data

Two real aCGH datasets were employed to test the hybrid CBS and eCBS on computation time and segmentation accuracy. Ten unpublished breast cancer aCGH arrays using the Agilent Human Genome CGH 105A platform with 105,072 probes, were analyzed. Probes with unknown positions or small signal-to-noise ratios (SNR *<*1) were filtered out, and more than 93,000 probes in each array were left for data segmentation. Another aCGH dataset from GSE9177 (NCBI/GEO, 11 aCGH profiles of human glioblastoma GBM using the Agilent 244A human CGH arrays) were also downloaded and processed for segmentation and performance comparison.

## Results

### Computation Time of the Hybrid CBS

In the breast cancer study with ten aCGH experiments, the percentage of time consumed by each critical step: candidate location, significance evaluation, and edge effect correction, are listed in Table 1. The results reveal that significance evaluation took at least 83% (93.7% on average) of the total time required to complete the segmentation process.

### The GEV Distribution Models Maximal-*t *Distribution Adequately

**Figure 3:**
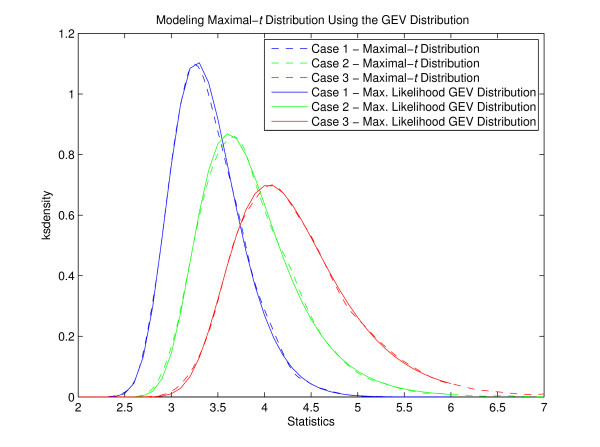
**The GEV distribution models maximal-***t ***distribution adequately**. The maximum likelihood GEV distribution fitted maximal-*t *distribution well. The dashed lines refer to the kernel smoothing density of the maximal-*t *statistics derived from random numbers, and the solid lines are the maximum likelihood GEV distribution. Three examples under different conditions are shown in the figure. Case 1: number of probes *N *= 250, skewness *sk *= 0, kurtosis *ku *= 3; Case 2: *N *= 400, *sk *= 0.5, *ku *= 4; and Case 3: *N *= 550, *sk *= 1, *ku *= 5.

To demonstrate whether the GEV distribution can model maximal-*t *distribution well, we generated 10,000 random datasets under different distributions controlled by skewness and kurtosis. The generated maximal-*t *distribution was then fitted by maximum likelihood method with the GEV distribution. Figure 3 shows examples of maximal-*t *distribution and the fitted GEV distribution under three different conditions: (1) normally distributed random data with 250 probes; (2) slightly skewed and heavy-tailed data with 400 probes; and (3) severely skewed and heavy-tailed data with 550 probes. The solid lines refer to the modeling GEV distribution while the dashed lines refer to the maximal-*t *distribution. The results clearly demonstrate that the GEV distribution can adequately model maximal-*t *distribution.

### The eXtreme Model Content Changes Smoothly

**Figure 4:**
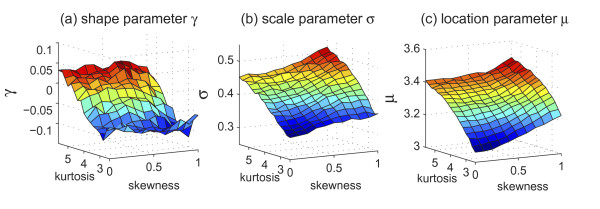
**The lookup tables in the eXtreme model**. (a) The GEV parameter *γ *spanned by the skewness and kurtosis when the number of probes *N *= 200. (b) The GEV parameter *σ *spanned by the skewness and kurtosis when *N *= 200. (c) The GEV parameter *μ *spanned by the skewness and kurtosis when *N *= 200.

For a specific number of probes *N*, the eXtreme model provides three 2-dimensional tables, indexed by skewness and kurtosis, for the GEV parameters, *γ*, *σ*, and *μ*, respectively. Figure 4 shows an example of the tables in the eXtreme model with *N *= 200. With two parameters, namely, the skewnesss and kurtosis (estimated from aCGH data), an approximation of maximal-*t *distribution in the form of the GEV parameters, *γ*, *σ*, and *μ*, can be quickly derived through the table lookup process. Additionally, as shown in the figure, since the eXtreme model content changes smoothly, linear interpretation can be properly applied to provide an approximation when the query parameters (*N*, *sk*, *ku*) do not fall on the grid.

### eCBS Performs Equivalently Comparing to the Hybrid CBS

The performance of the eCBS algorithm was tested according to the simulation models described early. Using the second simulation model, it is revealed that eCBS has a negligible effect on change-points detection from data with normal noise. In Additional File 1: Supplementary Table, the "Exact" column accounts for the cases (among 1,000 simulations) that the segmentation results exactly match the desired number (1 for edge and 2 for center) and locations of change-points. As shown in the table, eCBS performed as good as the hybrid CBS wherever change-points were located. In addition, eCBS outperformed when the aberration width was small (*k *= 2).

### eCBS Performs Adequately under Severely Skewed/Heavy-Tailed Conditions

**Figure 5:**
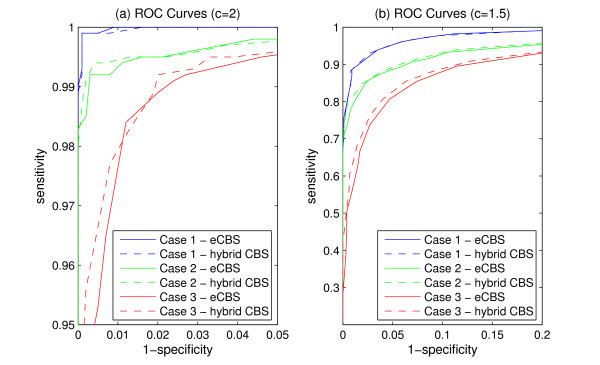
**The comparison of ROC curves between the hybrid CBS and eCBS**. The ROC curves obtained from the segmentation results of 10,000 simulated datasets. Each dataset contained 1,500 probes. Case 1 refers to the performance of analyzing normally distributed data with *sk *= 0, *ku *= 3. Case 2 refers to the performance of analyzing mildly skewed and heavy-tailed data with *sk *= 0.2, *ku *= 3.5. Case 3 refers to the performance of analyzing severely skewed and heavy-tailed data with *sk *= 0.5, *ku *= 4.0. (a) The performance of segmentation using the hybrid CBS and eCBS with large signal-to-noise ratios (SNR) (*c *= 2), and (b) with small SNR (*c *= 1.5).

The detection of genomic aberrations from data with skewed and heavy-tailed noises were further evaluated via simulation. ROC curves, obtained from an average of 10,000 simulations, were used to assess the effectiveness of both the hybrid CBS and eCBS. Three different conditions were studied in the performance comparison: normal distribution, mildly skewed/heavy-tailed distribution, and severely skewed/heavy-tailed distribution. The simulation results shown in Figure 5 reveal that the difference between the hybrid CBS and eCBS is minimal.

### eCBS Performs 4× to 20× Faster than the Hybrid CBS

To study the performance of eCBS in terms of the speed, ten breast cancer aCGH arrays were analyzed by both the hybrid CBS and eCBS, and the total time consumed on segmentation was compared and listed in Table 2. The time consumed on the pre-segmentation was taken into consideration when eCBS was applied. As shown in the table, it took eCBS much less computation time for segmenting: only about 10 seconds were required to analyze a dataset with eCBS, while on average, 68.68 seconds were needed for the hybrid CBS. The performance of eCBS in speed can be 4-fold faster, or even better. Taking arrays #28 and #45 for example, eCBS performed 20-fold faster than the hybrid CBS.

**Table 2:** Comparison of performance in speed using the hybrid CBS and eCBS - 1

	Time(sec.)		# of change-points		
		
Array	eCBS	hybrid CBS	eCBS	hybrid CBS	# of same detection
Array #10	11.30	82.56	66	55	53
Array #19	10.16	53.89	169	163	160
Array #22	6.45	77.94	101	110	97
Array #28	4.13	82.64	53	74	49
Array #42	4.02	74.44	31	51	31
Array #45	3.91	81.59	30	35	30
Array #48	2.16	29.18	1	1	1
Array #65	5.98	78.64	109	130	105
Array #72	12.90	56.83	106	103	95
Array #78	9.18	69.06	99	108	99

The computation time and segmentation results using the hybrid CBS and eCBS in a breast cancer study with 10 aCGH experiments. The time consumed in pre-segmentation was taken into consideration when eCBS was applied.

The segmentation results for eCBS were slightly different from those of the hybrid CBS. In samples #10, #19 and #72, eCBS detected more change-points than the hybrid CBS, while in the other experiments, eCBS detected fewer change-points. However, the segmentation results were mostly the same. The eCBS algorithm was also applied to a GSE9177 dataset of GBM samples. As shown in Table 3, eCBS performed four times faster than the hybrid CBS.

**Table 3:** Comparison of performance in speed using the hybrid CBS and eCBS - 2

	Time(sec.)		# of change-points		
		
Array	eCBS	hybrid CBS	eCBS	hybrid CBS	# of same detection
GSM231848	52.26	226.7	557	607	531
GSM231849	62.27	227.5	1208	1217	1118
GSM231850	78.48	203.8	2383	2577	2296
GSM231851	43.87	166.8	123	117	109
GSM231852	57.34	181.0	101	142	100
GSM231853	41.62	259.2	455	520	420
GSM231854	53.16	245.0	968	1010	920
GSM231855	49.91	249.1	1091	1174	1017
*GSM231856	32.09	297.8	291	625	291
GSM231857	91.86	223.2	2163	2116	2041
GSM231858	94.39	203.1	2476	2483	2376

The computation time and segmentation results using the hybrid CBS and eCBS in a human glioblastoma GBM study with 11 aCGH experiments. The time consumed in pre-segmentation was taken into consideration when eCBS was applied. Array GSM231856 is noted by a star (*) because the data is severely skewed and heavy-tailed. Thus, errors might occur.

## Discussion

Circular Binary Segmentation (CBS) performs consistently in detecting change-points and thus provides us a good framework for further improvements. The framework of CBS is mainly constituted of three steps: candidate location, significance evaluation, and edge effect correction. The first step, candidate location, locates candidate change-points by a maximal-*t *statistic. The second step, significance evaluation, approximates the significance of change-points by permutations or a hybrid method; the last step, edge effect correction, removes errors near the edges due to a circling process. Of these steps, significance evaluation was the major component that made the algorithm time-consuming. A time consumption study (shown in Table 1) on the hybrid CBS that involved analyzing ten breast cancer microarrays supported the above statement: among the ten experiments, up to 98% of time consumed was attributable to significance evaluation, in which permutations took the majority of the time for evaluating *p*-values, even when early stopping rules were applied. To improve the performance of the hybrid CBS in speed, we significantly reduce computation time using the eXtreme model.

The eXtreme model contains lookup tables which associates characteristics of aCGH data with the parameters of GEV distribution. The simulation, which applied the Pearson system to generate synthetic aCGH data, was done in advance rather than invoked on demand, such as the permutations for the hybrid CBS. Since maximal-*t *distribution is sensitive to heavy tails and outliers (shown in Figure 2), non-normal aCGH data distribution was considered for the simulation. Thousands of non-normal aCGH data under null hypothesis (without change-points) were simulated. The corresponding maximal-*t *distribution was then modeled by the GEV distribution, and the modeling results in the form of the GEV parameters, *γ*, *σ*, and *μ *, were saved in the eXtreme model (lookup tables).

Using the eXtreme model, maximal-*t *distribution can be approximated given the estimates of skewness (*sk*) and kurtosis (*ku*) from the aCGH data and the number of probes (*N*) under consideration. If the input parameters (*sk*, *ku*, *N*) fall right on the table grid, the output GEV parameters (*γ*, *σ*, *μ*) are directly derived through a table lookup process. Otherwise, since the table content changes smoothly (shown in Figure 4), linear interpolation from eight closest points in the 3-dimensional tables was applied. Through the table lookup process, maximal-*t *distribution and the subsequent approximation of the significance of change-points can be evaluated in *O*(1).

Deriving robust estimates of skewness and kurtosis from aCGH data is a critical step before using the eXtreme model, since estimating bias and variations may lead to incorrect approximations of maximal-*t *distribution and increase false positive or negative. In addition to the noise from microarray experiments, CNAs greatly increase the difficulties in estimating these parameters. Recognized what we needed here were estimates of skewness and kurtosis under null condition (the condition rarely exists because there are always CNAs within tumor samples), we selected a pre-segmentation step (see Additional File 1: Supplementary Materials) to rapidly pre-cut large regions of amplification or deletion. After removing the mean values of gain and loss segments, skewness and kurtosis can be accurately estimated.

Based on the eXtreme model, a novel algorithm, eCBS, has been developed. First, eCBS pre-segments data and estimates the skewness and kurtosis from the aCGH data. These estimates will be utilized later by the eXtreme model to provide approximations of maximal-*t *distribution. Once the estimates are provided, eCBS detects the maximal statistic, *T_max_*, and locates candidate change-points in a similar way operated by CBS. Please note that the maximal-*t *statistic is derived from the original aCGH data, not from the data after pre-segmentation. After candidate change-points are located, the *p*-value, or the significance of change-points, is evaluated based on the maximal-*t *distribution approximated using the eXtreme model. After edge effect correction further removes errors due to the circling process, change-points and the consequent subsegments are found. The process of finding change-points repeats iteratively until no more change-points can be detected. Rather than providing an upper bound or a lower bound of *p*-values, as implemented in the hybrid CBS, eCBS outperforms the hybrid CBS by approximating *p*-values for *T_max _*directly and reducing required permutations for significance evaluation.

## Conclusions

A novel algorithm, eCBS, was developed in this study, in which the significance of change-points is evaluated using the eXtreme model. The eXtreme model provides approximations of maximal-*t *distribution for hypothesis testing. With limited utilization of permutations, eCBS evaluates the significance of change-points through a table lookup process and achieves the best performance in speed. Via real aCGH data analysis and simulations, we showed that eCBS can perform as robustly as CBS, but with much less computation time. The eCBS algorithm with eXtreme model was implemented in an R package and is available from the supplementary website.

## Availability and Requirements

The eCBS algorithm was developed using Linux (version 2.6.11-1.1369 FC4smp) and R (version 2.8.0), and the source code is freely available at http://ntumaps.cgm.ntu.edu.tw/eCBSsupplementary. In order to execute properly, a compatible version of Linux operating system with R environment is required. Installation instructions are included in the manual (\eCBS\inst\doc\eCBS.pdf).

## Competing interests

The authors declare that they have no competing interests.

## Authors' contributions

FH, HC, and YC designed the eCBS algorithm and carried out the performance analysis. CH and ST collected the samples of breast cancer tissue. EC, MT and LL designed and participated in the coordination of microarray experiments. All authors collectively wrote the paper, read and approved the final manuscript.

## Supplementary Material

Additional file 1 **This additional file contains supplementary materials, supplementary figures and supplementary tables**. Click here for file
