# Supplementary material for: A model-based circular binary segmentation algorithm for the analysis of array CGH data
Source: BMC Res Notes. 2011 Oct 10;4:394. doi: 10.1186/1756-0500-4-394 (PMC3224564; doi:10.1186/1756-0500-4-394)
Supplement: Additional file 1 — This additional file contains supplementary materials, supplementary figures and supplementary tables. [file 1756-0500-4-394-S1.PDF]

Additional File 1 - A model-based circular binary segmentation algorithm for the analysis of array CGH data

Fang-Han Hsu[1], Hung-I H Chen[2], Mong-Hsun Tsai[4], Liang-Chuan Lai[5], Chi-Cheng Huang[1,6], Shih-Hsin Tu[6], Eric Y Chuang*[1], and Yidong Chen*[2,3]

[1]Graduate Institute of Biomedical Electronics and Bioinformatics, Department of Electrical Engineering, National Taiwan University, Taipei 106, Taiwan,
[2]Greehey Children's Cancer Research Institute, The University of Texas Health Science Center at San Antonio, San Antonio, TX 78229, USA,
[3]Department of Epidemiology and Biostatistics, The University of Texas Health Science Center at San Antonio, San Antonio, TX 78229, USA,
[4]Institute of Biotechnology, Center for Systems Biology and Bioinformatics, National Taiwan University, Taipei 106, Taiwan,
[5]Graduate Institute of Physiology, National Taiwan University, Taipei 100, Taiwan,
[6]Cathy General Hospital, Taipei 106, Taiwan

Contents

Comparison Platform ---------- 1
Typical Estimates of Skewness and Kurtosis from Real aCGH Data ---------- 2
Comparison of Performance between the Hybrid CBS and eCBS ---------- 2
The Validity of aCGH Data Simulation Using the Pearson System ---------- 4
Alternative Estimators of Skewness & Kurtosis ---------- 5

Supplementary Materials

Comparison Platform

Time consumption studies were made for comparing the algorithm performance in speed using the hybrid CBS and eCBS. The hardware for comparison is IBM xServer 235 with two Xeon 2.4GHz CPUs and 1G RAM. As for software, DNAcopy version 1.16 distributed through Bioconductor/R [http://www.bioconductor.org/] was installed. All parameters of CBS were set as default, and the imbedded smoothing function was used for removing outliers. If not special specified, significance threshold of maximal-*t* test was set as *p*-value < 0.01.

Typical Estimates of Skewness and Kurtosis from Real aCGH Data

Supplementary Figure 1 shows typical estimates of skewness and kurtosis from real aCGH data. These values are obtained from 10 breast cancer aCGH samples using the Agilent Human Genome CGH 105A and 11 human glioblastoma GBM aCGH samples (GSE9177) using the Agilent 244A human CGH arrays. As shown in the figure, aCGH data are typically skewed with -0.3 < skewness < 0.3 and heavy-tailed with 3.0 < kurtosis < 4.5.

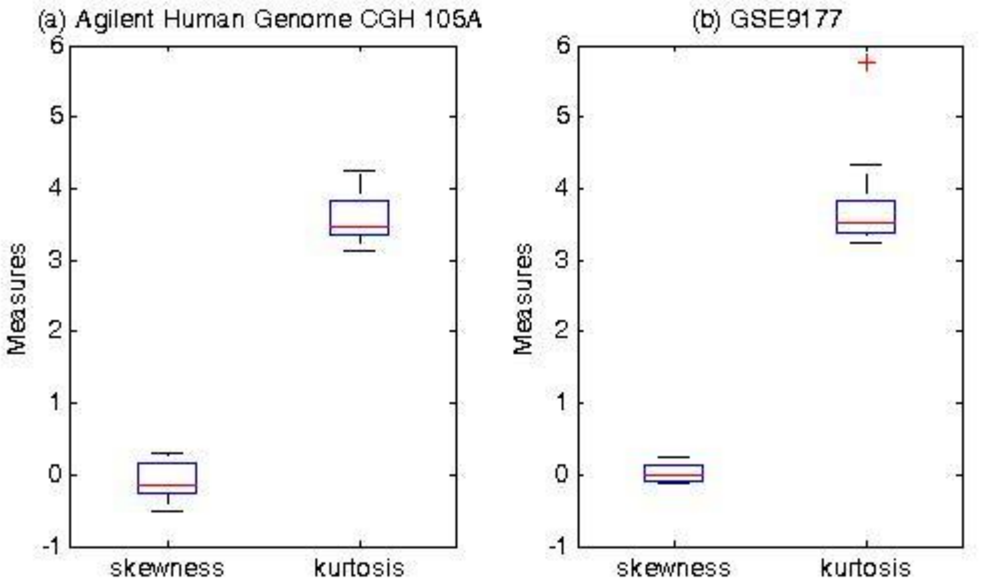

Supplementary Figure 1. Estimates of skewness and kurtosis on real data. These values are obtained from (a) 10 breast cancer aCGH samples using the Agilent Human Genome CGH 105A and (b) 11 human glioblastoma GBM aCGH samples (GSE9177) using the Agilent 244A human CGH arrays. Pre-segmentation was applied before evaluating the estimates; this is to avoid estimation bias due to extremely large values in the data.

Comparison of Performance between the Hybrid CBS and eCBS

The simulated data using the second model mentioned in the article contains 1,500 probes (N = 1,500) and one change-point near the edges or two change-points in the center of the chromosomes. The locations and amplitudes of change-points were controlled by $m_i = cvI$, where I is an indicator function, which equals 1 for segments between $\ell < x < (\ell + k)$ and 0 otherwise. Parameter k refers to the width of the variation, and ℓ refers to the location of the variation. Supplementary Table 1 shows the results.

Algorithm 1 : hybrid CBS (DNAcopy1.16)
Algorithm 2 : eCBS

			Change- points (edge)							Change- points (center)						
k	c	methods	Exact	0	1	2	3	4	>=5	Exact	0	1	2	3	4	>=5
2	2	hybrid CBS	**13**	974	17	9	0	0	0	**12**	971	0	29	0	0	0
		eCBS	**18**	968	22	10	0	0	0	**15**	966	0	33	0	1	0
	3	hybrid CBS	**179**	789	199	11	1	0	0	**152**	800	0	195	0	5	0
		eCBS	**218**	747	241	11	1	0	0	**180**	767	0	228	0	5	0
	4	hybrid CBS	**644**	316	668	7	8	1	0	**535**	399	0	593	0	8	0
		eCBS	**695**	269	719	9	2	1	0	**603**	333	0	656	0	11	0
3	2	hybrid CBS	**65**	898	91	11	0	0	0	**43**	909	0	90	0	1	0
		eCBS	**71**	888	99	13	0	0	0	**45**	897	0	101	0	2	0
	3	hybrid CBS	**393**	393	589	10	7	1	0	**449**	406	0	584	0	10	0
		eCBS	**377**	377	608	11	3	1	0	**457**	389	0	602	0	9	0
	4	hybrid CBS	**22**	22	963	5	10	0	0	**863**	25	0	958	4	13	0
		eCBS	**20**	20	972	4	3	0	0	**866**	27	0	956	0	17	0
4	2	hybrid CBS	**117**	801	187	11	1	0	0	**121**	762	0	234	0	4	0
		eCBS	**126**	786	201	12	1	0	0	**125**	748	0	248	0	4	0
	3	hybrid CBS	**715**	128	853	9	10	0	0	**599**	135	0	848	0	17	0
		eCBS	**726**	121	865	9	4	0	1	**604**	129	0	857	0	14	0
	4	hybrid CBS	**929**	1	983	5	11	0	0	**888**	2	0	980	0	18	0
		eCBS	**934**	1	988	6	4	0	1	**890**	2	0	982	0	16	0
5	2	hybrid CBS	**247**	623	362	11	3	1	0	**161**	627	0	365	0	8	0
		eCBS	**259**	600	383	13	2	1	1	**165**	612	0	378	0	10	0
	3	hybrid CBS	**802**	33	950	7	10	0	0	**667**	30	0	954	3	13	0
		eCBS	**807**	27	958	8	6	0	1	**668**	33	0	951	0	16	0
	4	hybrid CBS	**940**	0	986	4	10	0	0	**879**	0	0	984	0	16	0
		eCBS	**940**	0	986	3	10	0	1	**874**	0	0	978	0	22	0

Supplementary Table 1. The number of change-points detected by the hybrid CBS and eCBS. We applied these methods to 1,000 datasets; each of them contains 1,500 probes simulated from the normal distribution. The **Exact** columns count the number of cases in which the segmentation results exactly match the desired number (1 for edge and 2 for center) and locations of change-points. Here *k* is the width of the changed segment and *c* is the number of standard deviations between the two means. Each dataset had one elevated region ranging from 2 to 5 points, and the elevated region varied from 2 to 4 SDs above the mean. The cutoff of *p*-value for the simulation was 0.01.

The Validity of aCGH Data Simulation Using the Pearson System

In the study, the Pearson system was assumed sufficient to simulate a wide range of aCGH data under the null condition (no change-points). To assess the validity of our assumption, we simulated several datasets using the Pearson system and compared the distribution of simulated data to the distribution of real aCGH data using a two-sample Kolmogorov-Smirnov test (KS-test).

One of the 10 breast cancer and 11 glioblastoma GBM aCGH data indicated in Section Methods - Real aCGH Data was selected and pre-segmented; after the pre-segmentation process, the skewness and kurtosis of the array were estimated. Using the estimates of mean, standard deviation, skewness and kurtosis from the selected array as input parameters, we randomly generated 1,000 probes using a Matlab function *pearsrnd()* as the simulated data for hypothesis testing. Additionally, we randomly picked up 1,000 probes from the selected array after pre-segmentation. This set of probes is the real data for hypothesis testing. Now we have one simulated sample from the Pearson system and one real sample from the selected array. A two-sample KS-test with the null hypothesis - the two datasets under consideration are from the same continuous distribution - was applied. If the *p*-value is smaller than *alpha* = 0.01, we reject the null hypothesis.

Real Data	Size 1000	Size 100	Real Data	Size 1000	Size 100
Array #10	0	0	GSM231848	2	0
Array #19	0	1	GSM231849	1	1
Array #22	0	1	GSM231850	4	2
Array #28	1	1	GSM231851	2	0
Array #42	0	1	GSM231852	3	0
Array #45	2	1	GSM231853	1	1
Array #48	0	0	GSM231854	0	1
Array #65	0	0	GSM231855	0	1
Array #72	2	1	GSM231856	7	1
Array #78	0	1	GSM231857	0	2
			GSM231858	1	1

Supplementary Table 2. The number of times among 100 that the *p*-values of the two-sample KS-test are smaller than 0.01. Real data are drawn from the aCGH data labeled in the column *Real Data*, while the simulated data are drawn from the Pearson system with the parameters, mean, standard deviation, skewness, and kurtosis, being set as the same as the estimates derived from the corresponding array. The column *Size 1000* refers to the cases with 1,000 probes, and the column *Size 100* refers to the cases with 100 probes.

We repeated the process for 100 times per array and listed the number of times that the *p*-values are smaller than 0.01. Supplementary Table 2 shows the results. As shown in the table, whether data size is large (size = 1000) or small (size = 100), variables drawn from the real data and variables drawn from the Pearson system did not lead to statistically significant difference in distribution. This indicates that our assumption - the Pearson system can simulate aCGH data - is sound and most likely correct.

Alternative Estimators of Skewness & Kurtosis

To avoid estimation bias due to copy number alterations in data, we tried alternative estimators for the 2nd, 3rd, and 4th central moments as follows. Let r_1, r_2, ..., r_n denote independent and identically distributed (*i.i.d.*) random variables with $E[r_j] = \mu_r$. We are here interested in deriving the 2nd, 3rd, and 4th central moments. Assuming new random variables $\delta_{i+1,i}$, $\delta_{i+2,i}$, $\delta_{i+3,i}$, and $\delta_{i+4,i}$ as

$$\begin{aligned}\delta_{i+1,i} &= r_{i+1} - r_i,\\ \delta_{i+2,i} &= r_{i+2} - r_i,\\ \delta_{i+3,i} &= r_{i+3} - r_i,\\ \delta_{i+4,i} &= r_{i+4} - r_i,\end{aligned}$$

where $1 \le i \le n-1$ for $\delta_{i+1,i}$, $1 \le i \le n-2$ for $\delta_{i+2,i}$, $1 \le i \le n-3$ for $\delta_{i+3,i}$, and $1 \le i \le n-4$ for $\delta_{i+4,i}$. An unbiased estimator for the 2nd central moment $\hat{m}_2^r$ has been proposed and is given by

$$\begin{aligned}\hat{m}_2^r &= E[(r_j - \mu_r)^2]\\ &= E[r_j{}^2] - \mu_r{}^2\\ &= \frac{E[\delta_{i+1,i}{}^2]}{2}\\ &= \frac{\sigma^2_{\delta_{i+1,i}}}{2}\end{aligned} \quad . \qquad \text{(s1)}$$

Similarly, an unbiased estimator for the 3rd central moment $\hat{m}_3^r$ is proposed and given by

$$\begin{aligned}\hat{m}_3^r &= E[(r_j - \mu_r)^3]\\ &= E[r_j{}^3] - 3E[r_j{}^2]\mu_r + 2\mu_r{}^3\\ &= -E[\delta_{i+1,i}{}^2\delta_{i+2,i}]\\ &= \frac{-\sum_{i=1}^{n-2}\delta_{i+1,i}{}^2\delta_{i+2,i}}{n-2}\end{aligned} \quad , \qquad \text{(s2)}$$

and an unbiased estimator for the 4th central moment $\hat{m}_4^r$ is proposed and given by

$$\begin{aligned}\hat{m}_4^r &= E[(r_j-\mu_r)^4] \\ &= E[r_j^{\,4}]-4E[r_j^{\,3}]\mu_r+6E[r_j^{\,2}]\mu_r^{\,2}-3\mu_r^{\,4} \\ &= E[\delta_{i+1,i}\delta_{i+2,i}\delta_{i+3,i}\delta_{i+4,i}] \\ &= \frac{\sum_{i=1}^{n-4}\delta_{i+1,i}\delta_{i+2,i}\delta_{i+3,i}\delta_{i+4,i}}{n-4}\end{aligned} \quad . \qquad \text{(s3)}$$

Estimates of the skewness and kurtosis of aCGH data can theoretically be derived using $\hat{m}_2^r$, $\hat{m}_3^r$, and $\hat{m}_4^r$, which are given by

$$skewness = \hat{m}_3^r \big/ \hat{m}_2^{r\,3/2} ,$$

$$kurtosis = \hat{m}_4^r \big/ \hat{m}_2^{r\,2} .$$

The motivation of using the difference between neighboring probes, $\delta_{i+1,i}$, instead of the original data r_j, is from the observation (shown in Supplementary Figure 2) that bias due to copy number changes can be virtually removed. As shown in subplot (a), the original data contains a region of obvious copy number gain, while after conversion (from r_j to $\delta_{i+1,i}$), as shown in subplot (b), regional noise was converted to point noise.

Practically, the standard deviation of $\delta_{i+1,i}$, $\sigma_{\delta_{i+1,i}}$, can be easily achieved using the median of absolute deviation (MAD) to avoid the influence of point noise, or,

$$\sigma_{\delta_{i+1,i}} = 1.4785 \cdot MAD(\delta_{i+1,i}) .$$

Using the data conversion (from r_j to $\delta_{i+1,i}$) and the MAD method, we can obtain robust estimates of the 2nd central moment, $\hat{m}_2^r$. However, for the 3rd and 4th central moment, we cannot simply apply the mean or the MAD operators to get robust estimates. Multiple reasons are provided:

1) The input aCGH data, r_j, may not satisfy the assumption of independence completely. While we may get good estimates of standard deviation, the derivation of 3rd and 4th moments requires much stringent independence condition;

2) The MAD for estimating standard deviation requires a normal distribution of $\delta_{i+1,i}$. This is not the case for 3rd and 4th moments, where we cannot assume a symmetric distribution (skewness).

3) The mean operator is prone to point noise, whether the noise is due to the conversion from r_j to $\delta_{i+1,i}$, or due to the intrinsic noise from array measurement.

Our experience with real aCGH data indicates that the estimates provided by Eqs. (s1, s2, s3) were not robust enough due to above reasons. Thus, we applied the pre-segmentation process to get accurate estimates of skewness and kurtosis in the study.

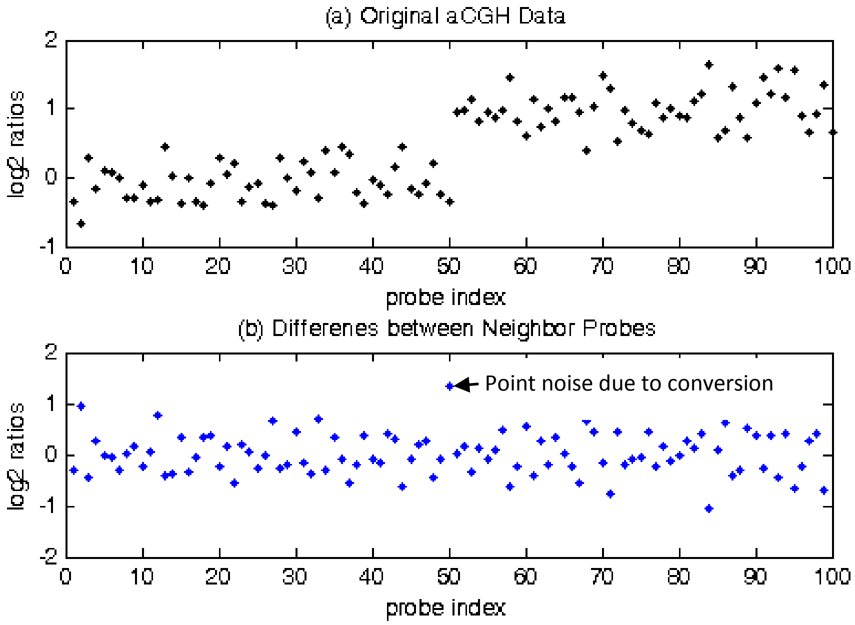

Supplementary Figure 2. Suppose a region (probe #51 to #100) of copy number gain lifts the sequential data by 1, (a) estimating central moments from the original aCGH data may lead to biased results due to regional noises from CNAs; (b) estimating central moments from differences between neighboring probes can result in minimized bias (point noise).
